# Metamaterial Broadband Absorber Induced by Synergistic Regulation of Temperature and Electric Field and Its Optical Switching Application

**DOI:** 10.3390/s24165430

**Published:** 2024-08-22

**Authors:** Rundong Yang, Yun Liu, Xiangfu Wang

**Affiliations:** 1 College of Electronic and Optical Engineering & College of Flexible Electronics (Future Technology), Nanjing University of Posts and Telecommunications, Nanjing 210023, China; b22100513@njupt.edu.cn (R.Y.); 1222025017@njupt.edu.cn (Y.L.); 2 Yunnan Key Laboratory of Electromagnetic Materials and Devices, Kunming 650091, China; 3 Key Laboratory of Radio Frequency and Micro-Nano Electronics of Jiangsu Province, Nanjing 210023, China

**Keywords:** broadband, bandwidth scalability, multi-layer, dual control

## Abstract

Nowadays, metamaterial absorbers still suffer from limited bandwidth, poor bandwidth scalability, and insufficient modulation depth. In order to solve this series of problems, we propose a metamaterial absorber based on graphene, VO_2_, gallium silver sulfide, and gold-silver alloy composites with dual-control modulation of temperature and electric field. Then we further investigate the optical switching performance of this absorber in this work. Our proposed metamaterial absorber has the advantages of broad absorption bandwidth, sufficient modulation depth, and good bandwidth scalability all together. Unlike the single inspired layer of previous designs, we innovatively adopted a multi-layer excitation structure, which can realize the purpose of absorption and bandwidth width regulation by a variety of means. Combined with the finite element analysis method, our proposed metamaterial absorber has excellent bandwidth scalability, which can be tuned from 2.7 THz bandwidth to 12.1 THz bandwidth by external electrothermal excitation. Meanwhile, the metamaterial absorber can also dynamically modulate the absorption from 3.8% to 99.8% at a wide incidence angle over the entire range of polarization angles, suggesting important potential applications in the field of optical switching in the terahertz range.

## 1. Introduction

Metamaterial absorber (MA) is a kind of optical device with wave-absorbing function based on metamaterials. With the first metamaterial perfect MA proposed by Landy et al. in 2008 [1], it has gradually been widely used in the fields of radar [2,3,4], optical switches [5,6], stealth material [7,8], and sensors [9,10]. Although the MAs proposed by people in recent years can realize the absorption of electromagnetic waves in almost various bands [11,12,13,14,15,16,17,18], they have the disadvantages of narrow absorption bandwidth and insufficient modulation depth, which can’t satisfy potential applications in optical devices, biotechnology, and other areas. Therefore, the methods to increase the absorption bandwidth and improve the tunability of the MAs have become an issue that has been discussed.

Preparing composite materials and changing the unit structure of the MAs are the conventional methods to improve the absorption bandwidth and increase the tunability. In 2021, Bai et al. proposed a tunable MA based on graphene floating grids, which has a bandwidth up to 2.597 THz. The MA’s maximum absorption can be tuned from 14.405% to 99.864% by controlling the Fermi energy level of the graphene surface [19]. In 2022, Yang et al. designed a tunable broadband terahertz MA based on the phase-change material VO_2_ with a bandwidth up to 3.88 THz, which can be dynamically tuned from 2.7% up to 98.9% by varying the conductivity of the VO_2_ [20]. In the same year, He et al. proposed a dual-controlled terahertz metamaterial absorber that achieves multidimensional modulation of broadband absorption with modulation depths up to 75% of the absorption rate by integrating photosensitive silicon into a graphene plasma [21]. In 2023, Zakir et al. utilized the graphene ring with different grooves to induce multiple plasma resonances with a bandwidth up to 2.4 THz, and the absorption characteristics can be tuned from 15% to 95% [22]. In 2023, Wang et al. proposed a photoelectrically excited terahertz hypersurface. By controlling the way of combining optically pumped and photosensitive silicon multilayers, the designed metasurface can work in four different states [23]. Although the absorption bandwidth and tunability of the above MAs have been improved, their bandwidths are still limited. Some scholars have realized the ultra-broadband absorption function of the MA, but the bandwidth scalability is poor and the modulation depth is shallow [24,25,26]. Similarly, the absorption bandwidths of the MAs with deep modulation depths are generally low, which makes it difficult to realize the function of the ultra-broadband absorption [27,28,29]. Therefore, how to design a MA that has the features of ultra-broadband, good bandwidth scalability, and deep modulation at the same time has become a major challenge.

To address the above problems, this paper proposes a multilayer structure broadband MA with dual-control adjustable temperature and electric field based on the composites of graphene, VO_2_, gallium-silver sulfide, and gold-silver alloy. In this paper, a multi-excitation layer structure is innovatively used, with graphene as the surface excitation layer and VO_2_, silver gallium sulfide, and gold-silver alloys as the intermediate excitation layers. We change the voltage applied to the graphene surface to change the surface conductivity of graphene using the change of the Fermi energy level to realize the electric control tunable [30,31,32]. By changing the ambient temperature, VO_2_ can realize the reversible transition from the dielectric state to the metallic state [33,34,35]. We again utilize this special property of VO_2_ to realize the temperature control. We performed calculations by means of finite element analysis. The results show that the MA proposed in this paper can realize the tuning of the absorption bandwidth from 2.7 THz to 12.1 THz with the absorption rate remaining above 90%. The MA has a modulation depth up to 96.2%, which can realize the tuning of the absorption rate from 3.8% to 99.8%, which indicates that it has very good optical switching performance.

## 2. Materials and Methods

**Figure 1:**
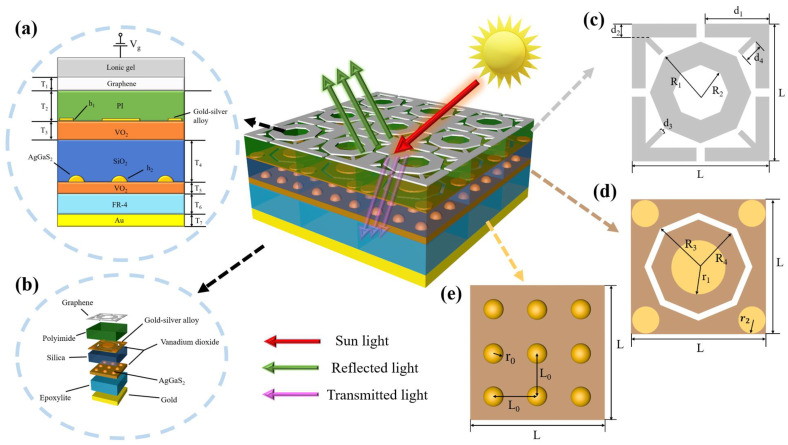
(**a**) Cross-section of the MA unit structure. (**b**) Structural decomposition of the MA. (**c**) Planar structure of the graphene layer of the MA. (**d**) Planar structure of the VO_2_-gold-silver alloy layer of the MA. (**e**) Planar structure of the MA VO_2_-gallium sulfide silver planar structure of the MA.

In this paper, we establish a multilayer structure MA based on VO_2_, graphene, and gallium silver-sulfur composite material shown in Figure 1. In the above model, the electromagnetic wave absorption phenomenon can be explained by the transmission line theory. When the wave (sunlight) is incident on the surface of the MA, part of the wave will be reflected to the free space (reflected light), and other part of the wave will be transmissive through the MA (transmitted light). Therefore, the absorption rate of the MA can be calculated by the following formula [36]:

(1) A=1−R−T

where *A* is the absorption, *R* is the reflection, and *T* is the transmittance. In this paper, we propose that the top of the MA is a layer of ionic gel, whose low absorption and high capacitance density properties will cause it to have almost no effect on the overall absorption spectrum. Figure 1a is a cross-section of the MA unit structure, showing how the external gate voltage *V_g_* is applied, where *V_DS_* is the drain-source voltage applied to ion gel. Figure 1b is the structural decomposition of the MA, whose bottom of the structure is gold. Moreover, the spacer layer is a lossy medium that is used to absorb the incoming electromagnetic waves. The structure has a total of three hypersurface layers, from top to bottom: graphene layer, VO_2_-gold-silver alloy layer, and VO_2_-gallium-sulfide-silver layer, respectively, which are designed as periodically arranged patterns to excite electromagnetic resonance so that the impedance of the MA matches the impedance of the free space. The geometrical parameters of the MA’s three excitation layers are shown in Figure 1c–e, where the period along both *x* and *y* directions is equal to *L* = 4.04 μm. The values of the dielectric constants of each spacer layer (PI, SiO_2_, FR-4) from top to bottom are *ε_r_*_1_ = 3.1, *ε_r_*_2_ = 3.9, *ε_r_*_3_ = 4.3, respectively [37,38], and the electrical conductivity of Au is *G* = 4.56 × 10^7^ S/m [39]. The following assumptions were made during the modeling process:

(1) It is assumed that the temperature of the external environment is the temperature of VO_2_.
(2) It is assumed that changes in the ambient temperature will not cause changes in the properties of materials other than VO_2_.
(3) It is assumed that the two VO_2_ layers have the same temperature.
(4) It is assumed that the voltage applied to the ionized gel will not cause changes in the properties of materials other than graphene.
(5) It is assumed that the MA does not generate heat after absorbing electromagnetic waves.
(6) It is assumed that the conductivity of the interband portion of the graphene surface is negligible.

In this paper, the Fermi energy levels on the graphene surface are changed by the change of bias voltage, the specific mathematical expressions of which are [40,41]:

(2) $$E_{f}=\hbar V_{f}\sqrt{\frac{\pi \varepsilon_{0}\varepsilon_{d}V_{g}}{es}}$$

where *V_f_* is the Fermi velocity, *s* is the distance between the positive and negative electrodes, *ε_0_* is the vacuum permittivity, *ε_d_* = 1.43 is the relative permittivity of ion gel, and *V_g_* is the applied bias voltage. Therefore, the conductivity of the graphene surface is affected by the Fermi energy level and different incident wave frequencies, which can be directly expressed by the Kubo formula [42,43]:

(3) $$\sigma_{\;}=\frac{{2e}^{2}k_{B}T}{{\pi \hbar }^{2}}\frac{i}{\left(\omega +i\tau^{-1}\right)}\ln\left[2\cosh\left(\frac{E_{f}}{2k_{B}T}\right)\right]\;+\frac{e^{2}}{4\hbar }\left[\frac{1}{2}+\frac{1}{\pi }\arctan\left(\frac{\hbar w-2E_{f}}{2k_{B}T}\right)-\frac{i}{2\pi }\ln\frac{{\left(\hbar \omega +2E_{f}\right)}^{2}}{{\left(\hbar \omega -2E_{f}\right)}^{2}+{\left(2k_{B}T\right)}^{2}}\right]$$

where *τ* is the carrier relaxation time, *e* is the amount of charge carried by electrons, *ℏ* is the reduced Planck’s constant, *k_B_* is Boltzmann’s constant, *T* is the thermodynamic temperature, and *E_f_* is the Fermi energy level of graphene. In the terahertz range at room temperature, the interband portion can be neglected. Therefore, the surface conductivity of graphene can be further expressed by the Drude-like formula [44]:

(4) $$\sigma_{\;}=\frac{e^{2}iE_{f}}{\hbar^{2}\pi \left(\omega +i\tau^{-1}\right)}$$

where we see that the conductivity of both the real and imaginary parts of the graphene surface increase with the chemical potential and decreases with the frequency. From this, we can then prove the correctness of the variation of graphene surface conductivity with the incident frequency as well as the Fermi energy level. In addition, for the relaxation time *τ*, we can realize the adjustment by chemical doping or external bias voltage [45]:

(5) $$\tau =\frac{\mu \hbar \sqrt{n\pi }}{eV_{f}}$$

where *μ* is the DC mobility and *n* is the carrier density.

For VO_2_, the Drude formula [46,47] can likewise be used to describe its dielectric constant in the terahertz range, which can be expressed as

(6) $$\varepsilon_{{VO}_{2}}=\varepsilon_{\infty }-\frac{{\omega_{p}}^{2}}{\omega \left(\omega +i\gamma \right)}$$

where *ω* is the frequency of the incident electromagnetic wave. *ε_∞_* is the high-frequency relative permittivity of VO_2_ with a value of 12, *γ* is the collision frequency, which usually takes the value of 5.57 × 10^13^ rad/s, and *ω_p_* is the plasma frequency associated with the conductivity of VO_2_, which can be expressed as

(7) $${\omega_{p}}^{2}=\frac{\sigma }{\sigma_{0}}\omega_{p_{0}}^{2}$$

where *σ*_0_ = 3 × 10^5^ S/m, *ω_p0_* = 1.45 × 10^5^ rad/s, and *σ_vo_*_2_ is the VO_2_ conductivity. With the change of external temperature, VO_2_ can realize the conversion between insulating and metallic states. At room temperature, VO_2_ presents an insulating state with a conductivity of 200 S/m, and the conductivity of VO_2_ can reach about 20,000 S/m by adjusting the temperature.

**Figure 2:**
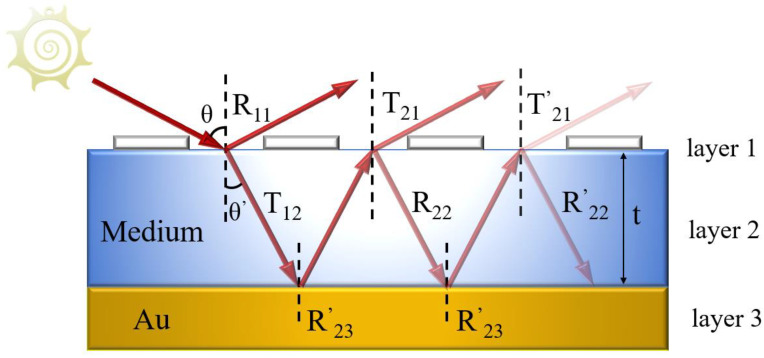
Multiple reflection interference equivalent model of MA.

We use the multiple reflection interference theory [22] (MRIT) for the validation of our simulation, which is shown in Figure 2.

First, terahertz waves are incident from air onto the surface of the MA. Then, part of the incident light is reflected by the graphene surface, and the other part is transmitted into the dielectric layer with reflection and transmission coefficients of *R*_11_ and *T*_21_, which can be expressed as:

(8) $$R_{11}=r_{11}e^{j\varphi_{11}}$$

(9) $$T_{21}=t_{21}e^{j\varphi_{21}}$$

The transmitted light will be completely reflected when it reaches the interface of the gold substrate with a reflection coefficient of *R*_23_, which can be expressed as:

(10) $$R_{23}=r_{23}e^{j\varphi_{23}}=-1$$

Then, as the light propagates to the surface of the structure, it will be reflected and transmitted again with reflection and transmission coefficients of *R*_22_ and *T*_12_, which can be expressed as

(11) $$R_{22}=r_{22}e^{j\varphi_{22}}$$

(12) $$T_{12}=r_{12}e^{j\varphi_{12}}$$

Finally, light undergoes multiple reflections and transmissions in the dielectric layer, and the final reflection efficiency *r* can be expressed as:

(13) $$r=R_{11}-\frac{T_{21}T_{12}\exp\left(2i\beta \right)}{1+R_{22}\exp\left(2i\beta \right)}$$

where phase shift *β* can be expressed as:

(14) β=nkt

where *n* is the refractive index of the dielectric layer, *k* is the number of angular waves in free space, and *t* is the thickness of the dielectric layer. Finally, the absorption rate can be expressed as

(15) $$A=1-{\left|r\right|}^{2}$$

where *A* is the absorption of the MA.

## 3. Results

Since our MA has fourfold rotational symmetry, the absorption spectrum of the MA is essentially polarization-independent, so in the following we consider only the case of TE incident light waves for our study. We use the method of finite element analysis for the simulation. We assume that the thickness of the graphene layer is *T*_1_ = 0.34 nm. As shown in Figure 1c, the geometric parameters of its pattern are *d*_1_ = 1.84 μm, *d*_2_ = 0.45 μm, *d*_3_ = 0.2 μm, *d*_4_ = 0.496 μm, *R*_1_ = 1.43 μm, and *R*_2_ = 0.55 μm. The thickness of the VO_2_-gold-silver alloy layer is *T*_3_ = 0.38 μm, in which the gold-silver alloy cylinder’s height is *h*_1_ = 0.2 μm. The geometrical parameters are *r*_1_ =1 μm, *r*_2_ =0.4 μm and the outer circle of the octagonal slit is *R*_3_ = 1.24 μm, *R*_4_ = 1.6 μm, respectively, in Figure 1d. Between the graphene layer and the VO_2_-gold-silver alloy layer is a layer of the PI dielectric with a thickness of *T*_2_ = 4.62 μm. Below the VO_2_-gold-silver alloy layer, we introduce a gallium-silver sulfur layer with a thickness of *T*_5_ = 0.8 μm and set *r_0_* = 0.4 μm, and *L_0_* = 1.2 μm as shown in Figure 1e. Between the VO_2_-gold-silver alloy layer and the gallium-silver sulfur layer is a silicon dioxide layer with a thickness of *T*_4_ = 1 μm. Finally, we choose gold with a thickness of *T*_7_ = 2.2 μm as the substrate layer. Between the substrate layer and the gallium-silver sulfur layer, we fill the FR-4 layer with a thickness of *T*_6_ = 0.69 μm. Moreover, since the width of graphene (*T*_1_) is only 0.34 nm, we model graphene as a two-dimensional planar material with zero thickness. Our proposed structure is experimentally realizable possibly. Graphene films can be grown using optimized chemical vapor deposition of a liquid precursor and determined to be monolayers by Raman measurements. Finally, the graphene film is mapped into the desired pattern using electron beam lithography, and the exposed areas can be etched away by oxygen plasma [48,49,50,51,52]. Then, by PLD deposition, we can obtain a VO_2_ layer and fabricate AgGaS_2_ using a crystal growth furnace [53,54]. Different dielectric layers can be obtained by deposition methods. Finally, we can realize the erection of multi-layer composite structures by cutting and gluing single layers [55].

### 3.1. MA Performance Analysis

**Figure 3:**
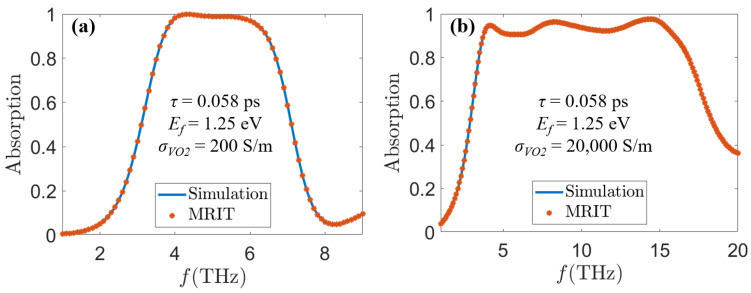
Relationship between simulated and theoretically calculated absorption spectra of the MA at (**a**) *τ* = 0.058 ps, *E_f_* = 1.25 eV, *σ_vo_*_2_ = 200 S/m, and (**b**) *τ* = 0.058 ps, *E_f_* = 1.25 eV, *σ_vo_*_2_ = 20,000 S/m.

As shown in Figure 3, with graphene surface *τ* = 0.058 ps, *E_f_* = 1.25 eV, and *σ_vo_*_2_= 200 S/m, our proposed MA can realize broadband absorption with more than 90% absorptivity from 3.7 THz to 6.4 THz, which have an average absorption of 97.6% and a bandwidth up to 2.7 THz. With graphene surface *τ* = 0.058 ps, *E_f_* = 1.25 eV, and *σ_vo_*_2_ = 20,000 S/m, our proposed MA can realize ultra-broadband absorption with more than 90% absorption from 3.8 THz to 15.9 THz, which have an average absorption of 93.9% and a bandwidth up to 12.1 THz. In order to prove the accuracy of our simulation results, we utilize multiple interference theory (MRIT) to verify the simulation results. In Figure 3, we can see that the absorption spectra derived with the MRIT algorithm fit well with those derived from our simulations, proving the accuracy of our simulation results.

**Figure 4:**
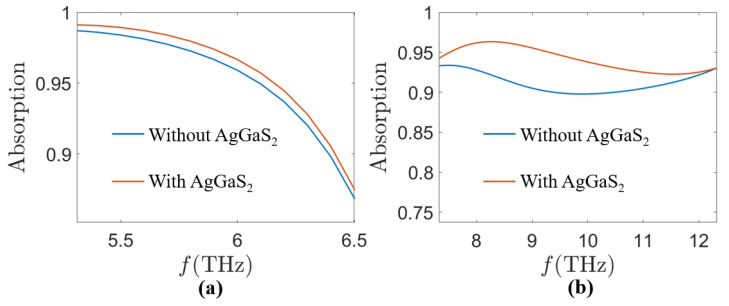
Absorption spectra of MA with and without AgGaS_2_ at (**a**) *τ* = 0.058 ps, *E_f_* = 1.25 eV, *σ_vo_*_2_ = 200 S/m, and (**b**) *τ* = 0.058 ps, *E_f_* = 1.25 eV, *σ_vo_*_2_ = 20,000 S/m.

In addition, the AgGaS_2_ bumps in SiO_2_ play an important role in modulating the absorption spectrum. To explore the effect of AgGaS_2_, we plotted the absorption spectra with and without AgGaS_2_, shown in Figure 4.

In the possession of AgGaS_2_, it is possible to slightly enhance the absorption from 5.5 THz to 6.5 THz for the MA in the case of *τ* = 0.058 ps, *E_f_* = 1.25 eV, *σ_vo_*_2_ = 200 S/m shown in Figure 4a. When the MA is at *τ* = 0.058 ps, *E_f_* = 1.25 eV, and *σ_vo_*_2_ = 20,000 S/m, AgGaS_2_ has a significant enhancement of the absorption in the 8 THz to 12 THz band of the absorption spectrum shown in Figure 4b.

**Figure 5:**
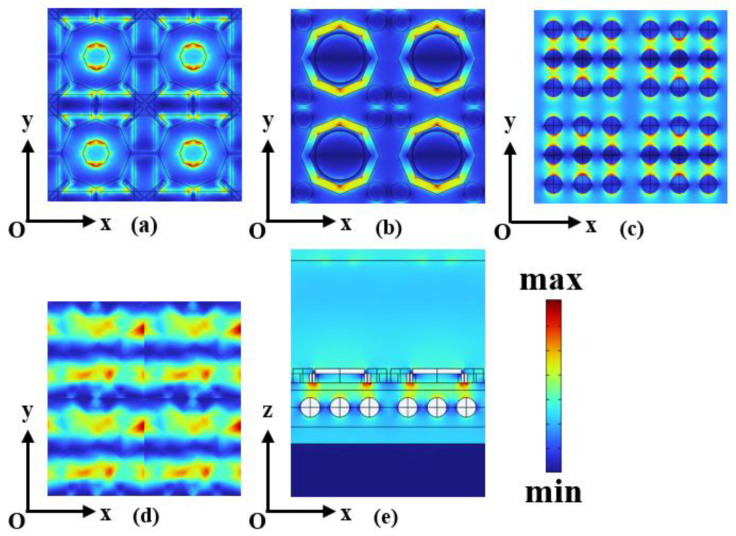
When MA graphene surface *τ* = 0.058 ps, *E_f_* = 1.25 eV, *σ_vo_*_2_ = 200 S/m, and 4.5 THz wave incidence (**a**) Graphene layer electric field strength *|E|* distribution, (**b**) VO_2_ layer electric field strength *|E|* distribution, (**c**) Gallium silver sulfur layer electric field strength *|E|* distribution, (**d**) Gold substrate surface electric field strength *|E|* distribution, and (**e**) *y* = 0 at the magnetic field strength *|H|* distribution in the *xoz* plane.

In order to understand the physical mechanism more clearly, we studied the distribution of electric field, surface current, and magnetic field corresponding to the MA’s surface. We find that the charge of the MA is mainly distributed at the graphene arms in the first layer shown in Figure 5a, the slit of VO_2_ in the second layer shown in Figure 5b, and the silver gallium sulfide sphere in the third layer shown in Figure 5c at *τ* = 0.058 ps, *E_f_* = 1.25 eV, and *σ_vo_*_2_ = 200 S/m. All three are due to the electric dipole resonance induced by the excitation of plasma-polarized excitons. Meanwhile, Figure 5d shows the electric field distribution excited at the gold substrate, and it can be seen that the charge is also distributed on the substrate surface of the MA.

Thus, the bottom current and the top current will form a current loop. In the process of forming the loop current, a magnetic polarizer will be formed, which will cause a strong magnetic response. The magnetic field distribution in the *xoz* plane is shown in Figure 5e, which shows that the magnetic field is not only confined around the graphene layer, VO_2_ layer, and gallium silver sulfide layer but also distributed in the FR-4, polyimide layer, and SiO_2_ layer. Meanwhile, the magnetic fields above and below the graphene pattern, VO_2_ layer, and gallium silver sulfide layer also imply the formation of magnetic resonance. Finally, the combined interaction of electrical and magnetic resonance leads to the formation of an absorption spectrum.

**Figure 6:**
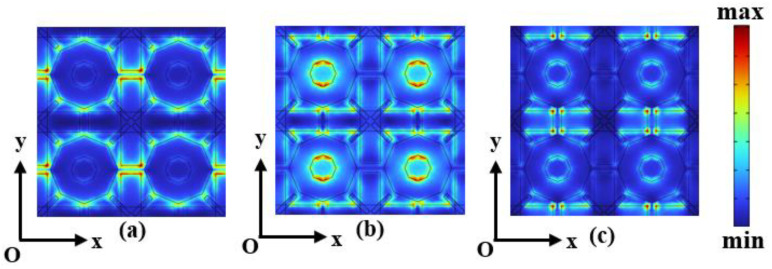
When MA graphene surface *τ* = 0.058 ps, *E_f_* = 1.25 eV, *σ_vo_*_2_ = 20,000 S/m, MA graphene layer surface electric field strength *|E|* distribution in the case of (**a**) 5 THz, (**b**) 10 THz, and (**c**) 15 THz incident wave.

Then, we adjust the temperature to realize the conversion of absorption spectra. For the convenience of the study, 5 THz, 10 THz, and 15 THz represent the low-frequency absorption band, the medium-frequency absorption band, and the high-frequency absorption band, respectively. Since the physical mechanisms of the VO_2_-gold-silver alloy layer and the gallium-silver sulfide layer exhibit roughly the same electric field distributions in the different frequency absorption bands, we have chosen the variation of the electric field distributions on the graphene surface as the main means of interpretation as shown in Figure 6. This phenomenon can be explained by the fact that different temperatures correspond to different conductivities of VO_2_. The change of conductivity affects the transmission and reflection of the incident wave, which in turn changes the electric field distribution on the graphene surface and finally achieves the purpose of changing the absorption spectrum.

**Figure 7:**
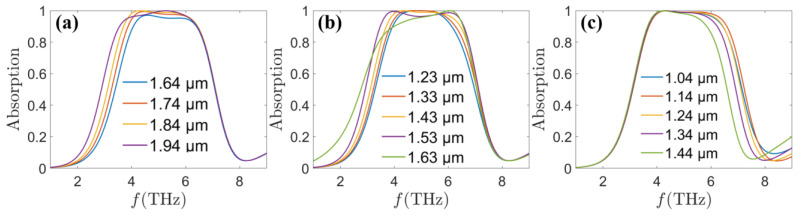
Absorption spectrum of the MA at *τ* = 0.058 ps, *E_f_* = 1.25 eV, *σ_vo_*_2_ = 200 S/m as a function of (**a**) *d*_1_, (**b**) *R*_1_, and (**c**) *R*_3_.

In addition, after a later study, it was found that the absorption characteristics of our proposed MA are also related to a series of geometrical parameters. The absorption spectrum of the MA as a function of *d*_1_ is shown in Figure 7a. With the increase of *d*_1_ from 1.64 μm to 1.94 μm, a slight red shift appears in the left absorption band. The reason attributed to the fact that with the increase of the length of the resonance cavity is increased, leading to the decrease of the resonance frequency. As shown in Figure 7b, when *R*_1_ increases from 1.23 μm to 1.63 μm, the absorption broadband range is slightly expanded because with the increase in the area of the octagonal shape of graphene, the distance between the octagonal shape of graphene and the surrounding pattern decreases. Therefore, coupling strength increases and the absorption broadband is expanded. When *R*_3_ increases from 1.04 μm to 1.44 μm shown in Figure 7c, the absorption band is slightly red-shifted, whose reason can be explained by the increase in the length of the VO_2_ slit resonance cavity.

**Figure 8:**
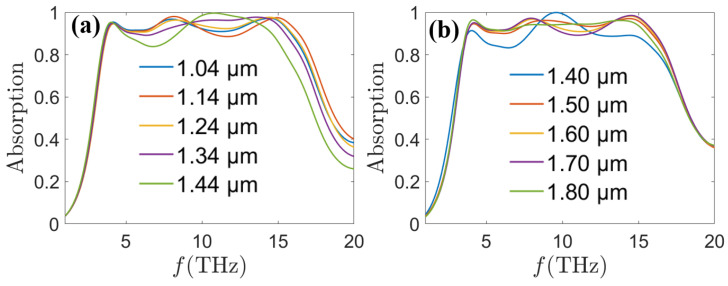
Absorption spectra of the MA at *τ* = 0.058 ps, *E_f_* = 1.25 eV, *σ_vo_*_2_ = 20,000 S/m as a function of (**a**) *R*_3_ and (**b**) *R*_4_.

When *R*_3_ increases from 1.04 μm to 1.14 μm shown in Figure 8a, the high-frequency absorption band is slightly blue-shifted, and this phenomenon can be explained by the reduction of the resonance cavity length. However, when *R*_3_ is further increased from 1.14 μm to 1.44 μm, the high-frequency absorption band begins to show a red shift, which may be due to the fact that with the further expansion of *R*_3_, the VO_2_ slit is gradually close to the gold-silver-alloy made of cylindrical columns, and the coupling relationship changes, so that a red shift will occur. As shown in Figure 8b, with the increase of *R*_4_ from 1.40 μm to 1.80 μm, the change in absorption is explained in terms of a change in the matching relationship between the surface impedance and the free space impedance. Based on the above analysis, we can conclude that small variations in geometric parameters do not lead to significant changes in the overall absorption performance, which helps to overcome the influence of manufacturing human as well as machine errors on the absorption performance. On the other hand, utilizing limited geometrical variations to adjust the absorption broadband according to different environments also provides us with more possibilities for practical production.

**Figure 9:**
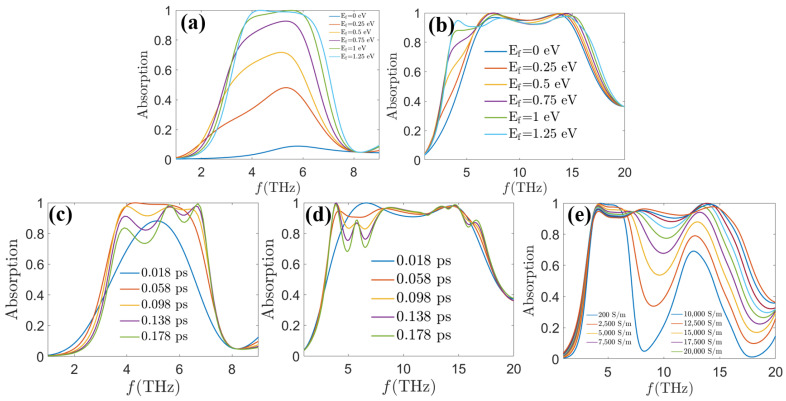
Absorption spectra of the MA as a function of (**a**) different Fermi energy levels when *τ* = 0.058 ps, *σ_vo_*_2_ = 200 S/m, (**b**) different Fermi energy levels when *τ* = 0.058 ps, *σ_vo_*_2_ = 20,000 S/m, (**c**) different relaxation times when *E_f_* = 1.25 eV, *σ_vo_*_2_ = 200 S/m, (**d**) different relaxation times when *E_f_* = 1.25 eV, *σ_vo_*_2_ = 20,000 S/m, and (**e**) different temperatures when *E_f_* = 1.25 eV, *τ* = 0.058 ps.

In addition, dynamic tuning of absorption properties is a major highlight of phase change materials such as graphene and VO_2_. Therefore, the effects of graphene surface Fermi energy levels, relaxation time, and ambient temperature on the absorption performance of this MA are also discussed in this paper. The results imply that, as shown in Figure 9a, the absorption peaks of the absorption spectra of the MA at *τ* = 0.058 ps, *E_f_* = 1.25 eV, and *σ_vo_*_2_ = 200 S/m gradually decrease with the decrease of the Fermi energy level. It may be caused by the weakening of the excitation intensity of the lattice plasmon resonance. When the Fermi energy level is 1.25 eV, the impedance matching condition can be well satisfied. When the MA is at *τ* = 0.058 ps, *E_f_* = 1.25 eV, and *σ_vo_*_2_ = 20,000 S/m, absorption spectra of different Fermi energy levels are given in Figure 9b. The low-frequency band absorption band has a significant decrease with the decrease of the Fermi energy level. The absorption of the rest of the frequency bands have different degrees of decreasing magnitude, but their absorption can always be maintained at a high level. Figure 9c,d show the absorption spectra under different graphene relaxation times. It is obvious that as the relaxation time increases from 0.018 ps to 0.178 ps, the absorption rates of the MA at *E_f_* = 1.25 eV, and σ*_vo_*_2_ = 200 S/m will gradually start to increase and then start to decrease again. When the MA is at *E_f_* = 1.25 eV and *σ_vo_*_2_ = 20,000 S/m, its average absorption decreases as *τ* increases from 0.018 ps to 0.178 ps. The reason for the increase in the absorption rate is that the contribution of the carriers to the plasma oscillations along the surface of graphene increases with the increase in the relaxation time. As the relaxation time increases further, the concentration of the carriers is close to saturation, and the number of carriers is too large, and most of the energy is reflected out, leading to a decrease in the absorption rate. Figure 9e shows the absorption spectra of the MA at different ambient temperatures. With the increase of ambient temperature, it is easy to see that the absorption rate as well as the absorption bandwidth of the MA undergo a significant change, and the tuning of the bandwidth length from 2.7 THz to 12.1 THz can be realized. The phenomenon can be explained by the change of the VO_2_ surface conductivity caused by the change of the electric resonance and the magnetic resonance, which also shows the good bandwidth scalability of our proposed MA.

**Figure 10:**
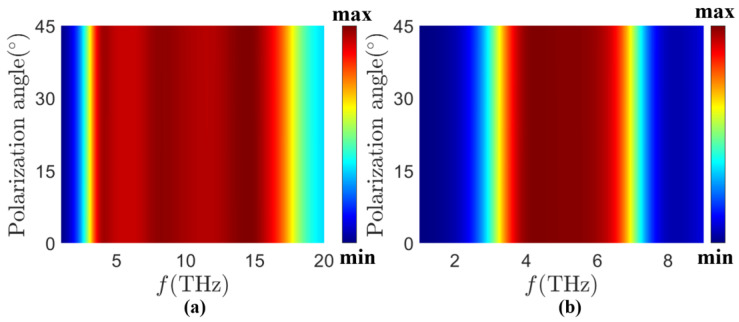
Absorption spectrum of the MA at different polarization angles for (**a**) *σ_vo_*_2_ = 20,000 S/m when *τ* = 0.058 ps, *E_f_* = 1.25 eV, and (**b**) *σ_vo_*_2_ = 200 S/m when *τ* = 0.058 ps, *E_f_* = 1.25 eV.

All of the results discussed previously are based on normally incident TE-polarized light. However, in a wider range of applications, the absorption at different polarization angles and variable angles of incidence can be equally worthy of discussion. The absorption properties under different polarization angles are shown in Figure 10. Due to the fourfold rotational symmetry of the structure, we only discuss the polarization angles in the range of 0° to 45°. It can be seen that the absorption band can still maintain an almost fixed position and bandwidth with a high absorption rate at different polarization angles, which implies that our proposed MA structure is polarization-insensitive. In addition, we have studied the absorption spectra of the MA at different incidence angles.

**Figure 11:**
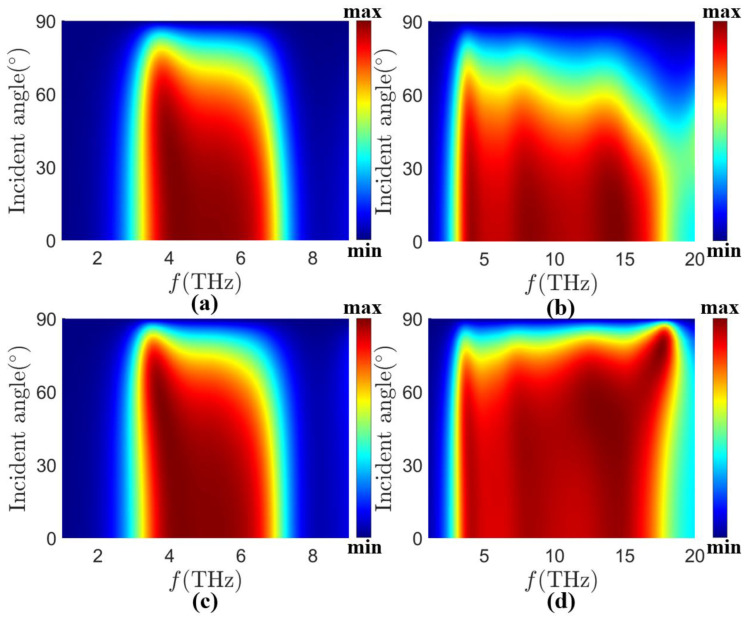
Absorption spectra of (**a**) TE wave at different incidence cases with MA at *τ* = 0.058 ps, *E_f_* = 1.25 eV, *σ_vo_*_2_ = 200 S/m, (**b**) TE wave at different incidence cases with MA at *τ* = 0.058 ps, *E_f_* = 1.25 eV, *σ_vo_*_2_ = 20,000 S/m, (**c**) TM wave at different incidence cases with MA at *τ* = 0.058 ps, *E_f_* = 1.25 eV, *σ_vo_*_2_ = 200 S/m, and (**d**) TE wave at different incidence cases with MA at *τ* = 0.058 ps, *E_f_* = 1.25 eV, *σ_vo_*_2_ = 20,000 S/m.

The absorption spectra of the MA at different incidence angles are shown in Figure 10. In the case of TE wave incidence, the MA can realize wide-angle absorption from 0° to 60° at *τ* = 0.058 ps, *E_f_* = 1.25 eV, and *σ_vo_*_2_ = 200 S/m given in Figure 11a, and from 0° to 40° at *τ* = 0.058 ps, *E_f_* = 1.25 eV, and *σ_vo_*_2_ = 20,000 S/m, given in Figure 11b. It should also be noted that the MA appears a slight red shift of the high-frequency absorption band when incidence at a larger incidence angle occurs at *τ* = 0.058 ps, *E_f_* = 1.25 eV, *σ_vo_*_2_ = 20,000 S/m. This is a result of the parasitic resonance that occurs at the larger incidence angle. In the case of TM wave incidence, the MA can achieve wide-angle absorption from 0° to 60° in both *τ* = 0.058 ps, *E_f_* = 1.25 eV, *σ_vo_*_2_ = 20,000 S/m, and *τ* = 0.058 ps, *E_f_* = 1.25 eV, *σ_vo_*_2_ = 200 S/m cases shown in Figure 11c,d. The absorption gradually decreases when the incidence angle is larger than 60°, which is due to the interaction between the incident light and the MA structure decreasing dramatically. Thus, the decrease in the absorptivity is easily understood. Moreover, we need to note that the high-frequency absorption band will be slightly blue-shifted in the case of larger incidence angle incidence at *τ* = 0.058 ps, *E_f_* = 1.25 eV, *σ_vo_*_2_ = 20,000 S/m. This can also be explained by the occurrence of parasitic resonance at larger incidence angles. In conclusion, our proposed MA has good absorption in both TE and TM wave incidence cases, which shows that our MA also has good wide-angle incidence performance. Due to the wide angle of incidence and polarization insensitivity of the structure, we believe that it has potential applications in many fields, such as multichannel absorption and multispectral detection techniques.

### 3.2. Optical Switch Performance Analysis

In order to further investigate the specific application of the MA proposed in this paper in the functional mode, we have investigated the MA optical switching performance. We utilize the performance of the MA at *τ* = 0.058 ps, *E_f_* = 1.25 eV, and *σ_vo_*_2_ = 200 S/m to realize the multi-switching function, where the absorption peaks and peaks and valleys correspond to the on and off states of the switch, respectively.

**Figure 12:**
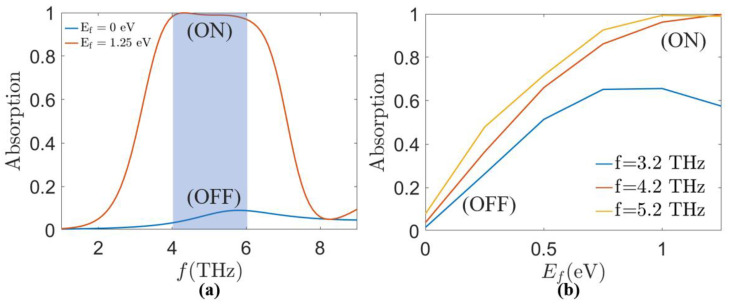
(**a**) Absorption spectra of Fermi energy levels *E_f_* = 0 eV and *E_f_* = 1.25 eV when the MA *τ* = 0.058 ps, *σ_vo_*_2_ = 200 S/m. (**b**) Absorption at 3.2 THz, 4.2 THz, and 5.2 THz for different *E_f_* when the MA *τ* = 0.058 ps, *σ_vo_*_2_ = 200 S/m.

In Figure 12a, we plotted the absorption spectra of the Fermi energy levels *E_f_* = 0 eV and *E_f_* = 1.25 eV. The Fermi energy level of the graphene surface for the “on” state is 1.25 eV, and that for the “off” state is 0 eV. To further demonstrate the light-switching nature of the MA, we extract the absorption at 3.2 THz, 4.2 THz, and 5.2 THz with different *E_f_* and show them in Figure 12b. When *E_f_* is increased from 0 eV to 1.25 eV, the absorption of the MA can increase from 3.8% to 99.8% at most with good coherence. This phenomenon can be explained by the enhancement of the excitation intensity of the lattice plasmon resonance. The performance of the optical switch can be described in terms of the modulation depth (*MDA*), which is expressed as [56].

(16) $$MDA=\frac{\left|A_{on}{-A}_{off}\right|}{A_{on}}\times 100\%$$

where *A_on_* and *A_off_* correspond to the absorption amplitude in the “on” and “off” states, respectively. The results show that the optical switch has an *MDA* of 96.2% at 4.2 THz with high modulation depth, indicating that the MA possesses excellent optical switching performance in the terahertz band. In order to show more the superiority of our proposed absorber, its absorption rate, absorption bandwidth (BW), fractional bandwidth (Fractional BW), and the absorption regulation are compared with those of previous absorbers proposed in the literature as shown in Table 1, which shows that the proposed absorber has the advantages of ultra-wideband and tunability.

## 4. Conclusions

In conclusion, we have designed a temperature-controlled, electrically dual-controlled tunable metamaterial MA based on graphene, VO_2_, and gallium-silver sulfide, which well solves the challenges of limited bandwidth, poor bandwidth scalability, and insufficient modulation depth that are usually faced by metamaterial MAs. Our MA simultaneously possesses the three advantages of ultra-broadband, good bandwidth scalability, and deep modulation depth. By means of electrothermal external excitation, the MA can realize the tuning of the absorption bandwidth from 2.7 THz to 12.1 THz, with the absorptivity maintained at more than 90%, and it has excellent ultra-broadband property and bandwidth scalability. For a wide range of incidence angles over the entire polarization angle range, the MA can accomplish the change of absorption from 3.8% to 99.8%, and the device has excellent optical switching performance with a modulation depth of up to 96.2%. In addition, our proposed MA is polarization-independent and operates well over a wide range of incidence angles, which can be considered an important component for terahertz modulation and filtering applications.

## Figures and Tables

**Table 1:** The comparison between references and our work.

Absorption	BW	Fractional BW	Absorption Regulation	Ref.
>90%	2.54 THz	0.80	14–99.9%	[25]
>90%	5.63 THz	1.00	1.2–97%	[26]
>90%	2.18 THz	0.69	32.8–99.9%	[27]
>80%	3.17 THz	0.75	33.51–99.99% or 76.31–99.99%	[28]
>90%	2.102 THz	0.75	-	[29]
>90%	2.7–12.1 THz	0.60–1.23	3.8–99.8%	Our work

## Data Availability

Data are contained within the article.

## References

[1] Landy N.I., Sajuyigbe S., Mock J.J., Smith D.R., Padilla W.J. (2008). Perfect metamaterial absorber. Phys. Rev. Lett..

[2] Yang H.H., Cao X.Y., Gao J., Liu T., Li S.J., Zhao Y., Yuan Z.D., Zhang H. (2013). Broadband low-RCS metamaterial absorber based on electromagnetic resonance separation. Phys. Rev. Lett..

[3] Liu T., Cao X.Y., Gao J., Zheng Q.R., Li W.Q., Yang H.H. (2013). RCS Reduction of Waveguide Slot Antenna with Metamaterial Absorber. IEEE Trans. Antennas Propag..

[4] Zou Y.K., Kong X.K., Xing L., Jiang S.L., Wang X.M., Wang H., Liu Z.M., Zhao Y.J., Bornemann J. (2022). A Slot Antenna Array With Reconfigurable RCS Using Liquid Absorber. IEEE Trans. Antenn. Propag..

[5] Tian X.Y., Qiu X.J., Li H., Lu J.J., Yang C.Y. (2024). Dynamically light-switched polarization-sensitive absorber based on semiconductor-incorporated metamaterial structure. Opt. Mater. Express.

[6] Shi Y.C., Chen X., Lou F., Chen Y.T., Yan M., Wosinski L.C., Qiu M. (2014). All-optical switching of silicon disk resonator based on photothermal effect in metal–insulator–metal absorber. Opt. Lett..

[7] Lu X.R., Chen J., Huang Y.Q., Wu Z.X., Zhang A.X. (2019). Design of Ultra-wideband and Transparent absorber based on Resistive Films. Appl. Comput. Electrom..

[8] Jing H.H., Wei Y.Q., Kang J.F., Song C.W., Deng H., Duan J.P., Qu Z., Wang J.Y., Zhang B.Z. (2023). An optically transparent flexible metasurface absorber with broadband radar absorption and low infrared emissivity. J. Phys. D Appl. Phys..

[9] Pang H.Z., Wang X., Wang J.L., Wang Z.L., Liu S.Y., Tian H.Q. (2021). Sensing characteristics of dual band terahertz metamaterial absorber sensor. Acta Phys. Sin..

[10] Li Y.L., An B.W., Jiang S.M., Gao J., Chen Y.L., Pan S.D. (2015). Plasmonic induced triple-band absorber for sensor application. Opt. Express.

[11] Zhang H.B., Deng L.W., Zhou P.H., Zhang L., Cheng D.M., Chen H.Y., Liang D.F., Deng L.J. (2013). Low frequency needlepoint-shape metamaterial absorber based on magnetic medium. J. Appl. Phys..

[12] Zhu W.R., Zhao X.P., Gong B.Y., Liu L.H., Su B. (2011). Optical metamaterial absorber based on leaf-shaped cells. Appl. Phys. A.

[13] Wang B.N., Koschny T., Soukoulis C.M. (2009). Wide-angle and polarization-independent chiral metamaterial absorber. Phys. Rev. B.

[14] Peng X.Y., Wang B., Lai S.M., Zhang D.H., Teng J.H. (2012). Ultrathin multi-band planar metamaterial absorber based on standing wave resonances. Opt. Express.

[15] Zhang Y.P., Zhao X.P., Bao S., Luo C.R. (2010). Dendritic metamaterial absorber based on the impedance matching. Acta Phys. Sin..

[16] Sohrab P., Atlasbaf Z. (2013). A Circuit Analog Absorber with Optimum Thickness and Response in X-Band. IEEE Antennas Wirel. Propag. Lett..

[17] Shi X.Z., Zang X.F., Wang Y.Q., Chen L., Cai B., Zhu Y.M. (2014). A polarization-independent broadband terahertz absorber. Appl. Phys. Lett..

[18] Lai S.F., Wu Y.H., Zhu X.B., Gu W.H., Wu W. (2017). An Optically Transparent Ultrabroadband Microwave Absorber. IEEE Photon. J..

[19] Bai J.J., Shen W., Shi J., Xu W., Zhang S.S., Chang S.J. (2021). A Non-Volatile Tunable Terahertz Metamaterial Absorber Using Graphene Floating Gate. Micromachines.

[20] Yang G.S., Yan F.P., Du X.M., Li T., Wang W., Lv Y.L., Zhou H., Hou Y.F. (2022). Tunable broadband terahertz metamaterial absorber based on vanadium dioxide. AIP Adv..

[21] He X.J., Wang D.J., Jiang J.X., Lu G.J., Yao Y.T., Gao Y.C., Yang Y.Q. (2022). Multidimensional manipulation of broadband absorption with dual-controlled terahertz metamaterial absorbers. Diam. Relat. Mater..

[22] Zakir S., Bilal R.M.H., Naveed M.A., Baqir M.A., Khan M.U.A., Ali M.M., Saeed M.A., Mehmood M.Q., Massoud Y. (2023). Polarization-Insensitive, Broadband, and Tunable Terahertz Absorber Using Slotted-Square Graphene Meta-Rings. IEEE Photon. J..

[23] Wang D.J., He X.J., Jiang J.X., Yao Y.T., Lu G.J. (2023). Photoelectrically-excited terahertz metasurface for switchable and tunable broadband propagation and polarization manipulations. Diam. Relat. Mater..

[24] Li T., Chen H., Zhang F.Q., Zhang J., Wang Z.L. (2022). An ultra-broadband terahertz absorber at high terahertz frequency. Opt. Quant. Electron..

[25] Fu M.X., Xia N., Duan Y.L., Zhou F., Li Y.S. (2024). Tunable broadband terahertz absorber based on graphene with bilayer hexagonal. AIP Adv..

[26] Pan Y.X., Dong J., Wang M., Luo H. (2024). Design of tunable ultra-wideband metasurface absorber with pixelated checkerboard pattern based on BGWO. Results Phys..

[27] Ri K.J., Kang R.J., Ri C.H. (2024). Tunable ultra-broadband terahertz metamaterial absorbers based on complementary split ring-shaped graphene. AIP Adv..

[28] Zhu Y.X., Niu H.J., Li Y.H., Lv T.G., Li H.F., Fan X.Y., Bai C.L. (2024). Tunable metamaterial broadband perfect absorber based on double-layer graphene nanofilm. Opt. Mater. Express.

[29] Weng X.H., Yan D.X., Qiu Y., Li X.J., Zhang L., Li J.N. (2024). Realization of multifunctional transformation based on the vanadium dioxide-assisted metamaterial structure. Phys. Chem. Chem. Phys..

[30] Huang Z.T., Jiang H.Y., Wang Z.Y., Qing Y.M., Li B.X. (2024). Thermally-Electrically Tunable Graphene-Based Guided-Mode Resonant Perfect Absorber. IEEE Photonics Technol. Lett..

[31] Patel S.K., Sorathiya V., Lavadiya S., Thomas L., Nguyen T.K., Dhasarathan V. (2020). Multi-layered Graphene Silica-Based Tunable Absorber for Infrared Wavelength Based on Circuit Theory Approach. Plasmonics.

[32] Du X.M., Yan F.P., Wang W., Tan S.Y., Zhang L.N., Bai Z.Y., Zhou H., Hou Y.F. (2020). Graphene-embedded broadband tunable metamaterial absorber in terahertz band. J. Opt..

[33] Dicken M.J., Aydin K., Pryce I.M., Sweatlock L.A., Boyd E.M., Walavalkar S., Ma J., Atwater H.A. (2009). Frequency tunable near-infrared metamaterials based on VO_2_ phase transition. Opt. Express.

[34] Kim Y., Wu P.C., Sokhoyan R., Mauser K., Glaudell R., Shirmanesh G.K., Atwater H.A. (2019). Phase modulation with electrically tunable vanadium dioxide phase-change metasurfaces. Nano Lett..

[35] Liu H., Lu J., Wang X.R. (2018). Metamaterials based on the phase transition of VO_2_. Nanotechnology.

[36] Liu X., Xia F., Wang M., Liang J., Yun M. (2023). Working Mechanism and Progress of Electromagnetic Metamaterial Perfect Absorber. Photonics.

[37] Tian X.L., Zhang H.F., Kong X.R. (2020). An Angle-Insensitive Metamaterial Absorber Based on the Gravity Field Regulation. Plasmonics.

[38] Qi L., Liu C., Shah S.M.A. (2019). A broad dual-band switchable graphene-based terahertz metamaterial absorber. Carbon.

[39] Li J.S., Yan D.X., Sun J.Z. (2019). Flexible dual-band all-graphene dielectric terahertz absorber. Opt. Mater. Express.

[40] Lv Y.S., Tian J.P., Yang R.C. (2021). Multiband tunable perfect metamaterial absorber realized by different graphene patterns. J. Opt. Soc. Am. B.

[41] Gómez-Díaz J.S., Perruisseau-Carrier J. (2013). Graphene-based plasmonic switches at near infrared frequencies. Opt. Express.

[42] Tang X., Jia H.D., Liu L.Y., Li M., Wu D., Zhou K., Li P., Tian L.Y., Yang D.L., Wang W.J. (2023). A Tunable Terahertz Absorber Based on Double-Layer Patterned Graphene Metamaterials. Materlals.

[43] Yao Y., Kats M.A., Genevet P. (2013). Broad Electrical Tuning of Graphene-Loaded Plasmonic Antennas. Nano Lett..

[44] Gupta S.K., Basu P.K. (2022). Tunability in Graphene Based Metamaterial Absorber Structures in Mid-Infrared Region. IEEE Photonics J..

[45] Zhang J.G., Tian J.P., Li L. (2018). A dual-band tunable metamaterial near-unity absorber composed of periodic cross and disk graphene arrays. IEEE Photon. J..

[46] Zhang M., Song Z.Y. (2021). Switchable terahertz metamaterial absorber with broadband absorption and multiband absorption. Opt. Express.

[47] Hu B.J., Huang M., Yang L., Zhao J.Y. (2023). Terahertz dual-tunable absorber based on hybrid gold-graphene-strontium titanate-vanadium dioxide configuration. Opt. Mater. Express.

[48] Jo G., Choe M., Cho C.Y., Kim J.H., Park W., Lee S., Hong W.K., Kim T.W., Park S.J., Hong B.H. (2010). Large-scale patterned multilayer graphene films as transparent conducting electrodes for GaN light-emitting diodes. Nanotechnology.

[49] Fang Z.Y., Wang Y.M., Schlather A.E., Liu Z., Ajayan P.M., de Abajo F.J.G., Nordlander P., Zhu Z., Halas N.J. (2014). Active tunable absorption enhancement with graphene nanodisk arrays. Nano Lett..

[50] Zhuo Q.Q., Wang Q., Zhang Y.P., Zhang D., Li Q.L., Gao C.H., Sun Y.Q., Ding L., Sun Q.J., Wang S.D. (2015). Transfer-free synthesis of doped and patterned graphene films. ACS Nano.

[51] Wang L.Z., Zhang J., Liu N., Wang Y.K., Hu P.A., Wang Z.L. (2016). Fast patterned graphene ribbons via soft–lithography. Procedia CIRP.

[52] Ye L.F., Chen X.E., Zhu C.H., Li W.W., Zhang Y. (2020). Switchable broadband terahertz spatial modulators based on patterned graphene and vanadium dioxide. Opt. Express.

[53] Zeng W., Chen N., Xie W.G. (2020). Research progress on the preparation methods for VO_2_ nanoparticles and their application in smart windows. CrystEngComm.

[54] Chen B.J., Zhu S.F., Zhao B.J., Zhang J.J., Huang Y., Li M., Liu J., Tan B., Wang R.L., He Z.Y. (2006). Growth of AgGaS_2_ single crystals by modified furnace. J. Cryst. Growth.

[55] Wang X., Chen X.M., He Q.Y., Hui Y.Z., Xu C.F., Wang B.C., Shan F.H., Zhang J., Shao J.Y. (2024). Bidirectional, Multilayer MXene/Polyimide Aerogels for Ultra-Broadband Microwave Absorption. Adv. Mater..

[56] Wu Y.F., Cai P.G., Nie Q.M., Tang C.J., Liu F.X., Zhu M.W. (2023). Ultra-narrowband, electrically switchable, and high-efficiency absorption in monolayer graphene resulting from lattice plasmon resonance. Results Phys..

